# Great expectations

**DOI:** 10.1186/1546-0096-9-S1-P316

**Published:** 2011-09-14

**Authors:** Noora Paruys

**Affiliations:** 1 Orka, GENK, Belgium

## 

First of all, I would like to thank you for giving me the opportunity to bring to you a point of view on juvenile arthritis which might seem somewhat strange in the setting of this congress. I represent an organization with a very particular point of view, the one of parents of children who suffer from this disease.

Allow me to focus on what the expectations of those patients ànd their parents and families are.

## Great Expectations

You probably know the 19^th^ century novel by Charles Dickens. This novel is what the Germans call ‘ein Bildungsroman’, a story of personal development which concentrates on the spiritual, moral, psychological and social development and growth from childhood to maturity. “Great Expectations” is a novel about a boy becoming a man through a disturbed and difficult childhood.

In fact, the theme of this novel is exactly the same theme in the minds of parents with an child, suffering from a chronical disease. And it should be very clear that these parents – like all parents – have expectations for their children, great expectations.

In October 2006 two mothers initiated in Flanders an organization for parents with children with juvenile arthritis. These parents are telling a story about being a parent with growing expectations, evolving needs on different levels.

On the first level the perspective broadens from an attempt to ease pain to an attempt tot raise a child with a disturbed childhood. The more parents discover about the disease of their child, the more their concerns evolve into other than pure medical matters. Education, character building, social abilities, finding a job, a partner, a meaning in life, getting settled…

The second level of growing needs is the one about information and communication. The more parents learn about this disease, the more information is needed in order to take appropriate responsibility and action. In that context the frequency, depth and quality of the communication between parents on the one hand and the medical and paramedical staff on the other, is of great importance, although their languages often seem incompatible, living in separate worlds as they are. Parents and doctors should be partners in helping and guiding a sick child. And again, it shows that communication is full traffic in two directions between partners with equal abilities, the one as a doctor, the other as a parent, talking about the same child with respect for ànd using each others expertise, responsibility and position.

And last but not least, there is the way in which society looks upon a child that is chronically ill. The environment has a lot of pity, eagerness to protect this child from pain, from not being understood and from so many other threats. But this childs illness is not lifethreatening. So, there is a future and the main task of the environment is not to pity this child but to empower it to become an adult and strong member of society.

And this task, this process of empowerment is a shared responsibility and probably the major challenge parents and doctors have to face together.

